# Influence of heat flow control on dynamical spin injection in CoFeB/Pt/CoFeB trilayer

**DOI:** 10.1038/s41598-022-06784-6

**Published:** 2022-03-02

**Authors:** Sora Obinata, Riku Iimori, Kohei Ohnishi, Takashi Kimura

**Affiliations:** Department of Physics, Kyushu University, 744 Motooka, Fukuoka, 819-0395 Japan

**Keywords:** Spintronics, Magnetic devices

## Abstract

A dynamical spin injection based on the ferromagnetic resonance in a ferromagnetic/nonmagnetic bi-layered structure, is a powerful mean for generating and manipulating the spin current. Although the mechanism of the dynamical spin injection is mainly attributed to the spin pumping, the detailed mechanism and the quantitative understanding for related phenomena are still controversial. As an another important contribution to the dynamical spin injection, the heating effect due to the resonant precessional motion of the magnetization is pointed out recently. In order to quantify the contribution from the heating effect, we here investigate the dynamical spin injection in a CoFeB/Pt/CoFeB trilayer. Although the contribution from the spin pumping diminishes because of the symmetric spin injection from the upper and lower interfaces, a significant inverse spin Hall voltage has been clearly observed. We show that the observed voltage can be quantitatively understood by the thermal spin injection due to a heating effect during the ferromagnetic resonance. A proper combination between the spin pumping and the heat-flow control in the multi-layered system is a key for the efficient dynamical spin injection.

## Introduction

Spintronic devices consisting of ferro-magnet (FM) /non-magnet (NM) hybrid structures have attracted much attention since the discovery of the spin-dependent transports^1^. It is well known that a spin current, a flow of spin angular momentum, plays a significant role in operations of the spintronic devices^2^. This is because the spin current can be utilized for manipulating the magnetization as well as for detecting the magnetization direction^3,4^. The spin current is, in general, created by applying an electric field across the FM/NM interface, resulting in injecting the spin-polarized electrons from the FM into the NM^5–8^. This is known as electrical spin injection, which is a widely utilized for generating the spin current because of its high selectivity and flexibility. In addition, since the Seebeck coefficient in the ferromagnet also shows the spin dependence, the spin current can be generated by using the temperature gradient across the FM/NM interface, instead of the electric field^9–11^. This is known as the thermal spin injection, where the generation efficiency often shows the significant enhancement in several ferromagnetic metals depending on its band structure^12–14^.

Apart from the aforementioned statical spin injection, the dynamical spin injection based on a ferromagnetic resonance (FMR) is also an attractive mean for generating and manipulating the spin current^15–17^. This is because the FMR can be induced by microwave irradiation without electrical connection, leading to wireless spintronics^18,19^. So far, the mechanism of the dynamical spin injection is attributed to the spin pumping, where the excess spins in the FM due to the FMR spill out into the adjacent nonmagnetic heavy metal (HM)^20–22^. On the other hand, the resonant precessional motion of the magnetization is known to yield the heating effect through the magnon-phonon interaction^23–26^. This FMR heating effect produces the temperature gradient across the FM/NM interface, resulting in the thermal spin injection^12,14^. Since the temperature change due to the FMR heating exceeds 10 K, it could be the significant contribution on the dynamical spin injection^19,24^. However, since the dynamical spin injection is mainly evaluated by measuring the inverse spin Hall effect (ISHE), it is difficult to distinguish the dominant contribution in the dynamical spin injection. Moreover, the proper combination between the spin pumping and the thermal spin injection will enhance the dynamical spin injection efficiency significantly. Therefore, it is essential to understand how large the thermal spin injection contributes to the dynamical spin injection. We have developed an effective device structure for the quantitative evaluation of the dynamical spin injection^27^. In this method, the spin-rectified voltage caused by the charge current directly flowing in the ferromagnetic layer can be minimized by using a thick Cu electrode, leading to the quantitative analysis of the dynamical spin injection. Very recently, we showed that a CoFeB/Pt/CoFeB trilayered structure is effective for the investigation of a dynamical spin injection^28^. In the present paper, by extending our evaluation method, we investigate the contribution of the thermal spin injection in dynamical spin injection. Especially, by controlling the temperature gradient due to FMR heating effect, we clarify the importance of the thermal spin injection.

## Experimental procedure

**Figure 1:**
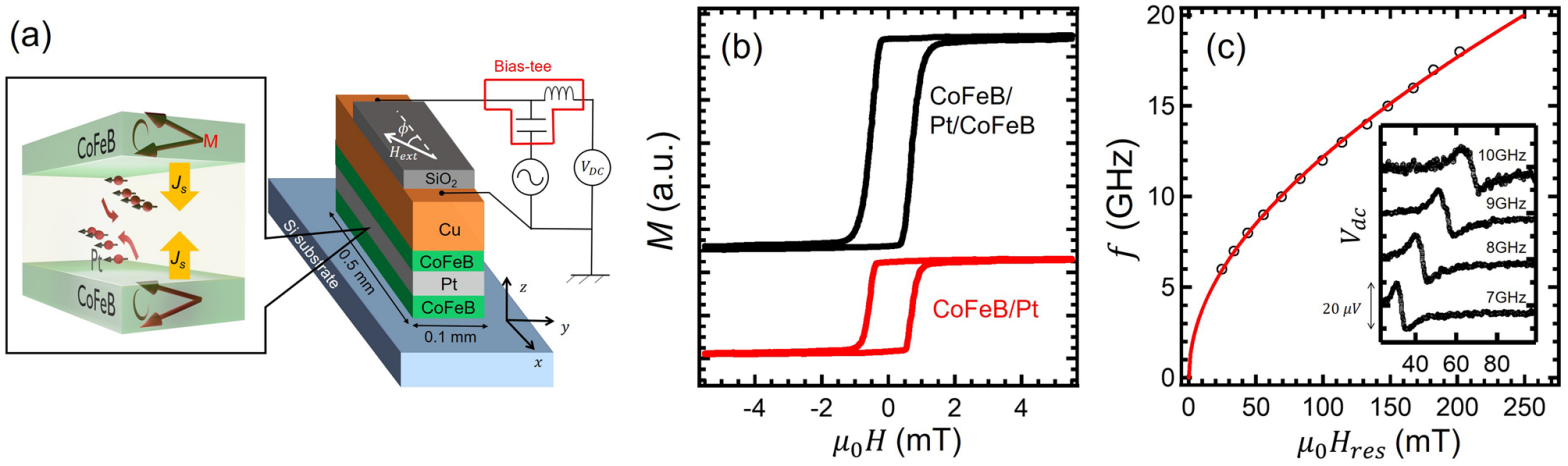
(**a**) Schematic illustration of the fabricated device based on CoFeB/Pt/CoFeB trilayer together with the probe configuration for RF measurements. The inset shows a conceptual image of the dynamical spin injection in the trilayer. (**b**) Room temperature M-H curves measured by VSM for CoFeB/Pt bilayer and CoFeB/Pt/CoFeB trilayer. (**c**) Relationship between the resonant magnetic field $H_\mathrm{res}$ and the microwave frequency *f*. The solid line represents the fitting curve using Kittel’s equation. The inset shows the field dependence of dc voltage under the microwave signal with various frequencies observed in the trilayered sample.

The sample used for the present study consist of a CoFeB/Pt/CoFeB tri-layered structure with a Cu electrode, as schematically shown in Fig. 1a. Here, the trilayer has been grown by a magnetron sputtering system with the base pressure of $8 \times 10^{-6}$Pa on a non-doped floating-zone Si substrate. The thickness of the Pt layer (the upper and bottom CoFeB layers) is 5 nm (10 nm). Then, a 100-nm-thick Cu electrode was entirely deposited on the trilayer. Here, the multi-layered film was patterned to 100-$\mu$m-width and 500-$\mu$m-length rectangular-strip shape by the lithography. In order to avoid the surface oxidation, a 10-nm-thick SiO$_2$ capping layer was sputtered on the top of the Cu electrode.

The present CoFeB/Pt/CoFeB tri-layered structure is effective for acquiring the contribution of the thermal spin injection selectively because of the following reason. When the upper and lower CoFeB layers have the same magnetic property, the FMR can be excited simultaneously. In such a situation, the dynamical spin injections occur both from the upper CoFeB/Pt and lower Pt/CoFeB interfaces^15–17,19^. As schematically shown in Fig. 1a, the flowing direction of the spin current injected from the upper and lower interfaces are opposite each other while the polarizations are the same direction. Since the spin pumping is the interfacial effect^20–22^, the magnitude of the spin injection due to the spin pumping from both sides are almost same. Therefore, the contribution of the spin pumping is expected to diminish in the present trilayer. On the other hand, from the viewpoint of the thermal spin injection, since the magnetic trilayer is sandwiched by the Cu electrode and the Si substrate, the temperature distribution becomes asymmetric^12,14^. In this case, the magnitude of the thermal spin injections from the upper and lower interfaces should be different. This leads to generate the detectable ISHE voltage. Thus, we are able to extract the contribution of the thermal spin injection due to the FMR heating effect effectively.

## Results and discussions

First, we evaluate the static magnetic property of the trilayed film by using a vibrating-sample magnetometer (VSM). As show in Fig. 1b, the magnetization curve exhibits a single sharp switching with the coercivity as low as 5 Oe. For the comparison, we also show the magnetization curve for the CoFeB/Pt bilayer. We confirm the magnetization curve similarly to the trilayed film with almost the same coercivity. These results indicate that the upper and lower CoFeB films have the same magnetic property with the typical characteristic of soft magnetic materials.

We then evaluate the dynamic property of the present trilayer by measuring the dc voltage along the longitudinal direction under the RF current injection, as schematically shown in Fig. 1a. Here, the microwave power is $-5$ dBm and the microwave frequency range is varied from 6 GHz to 18 GHz. The inset of Fig. 1c shows the representative spectra observed in the present sample for various frequencies measured at an angle $\phi = 45$ deg with respect to the current direction. The shapes of the spectra are almost asymmetric, suggesting that the ISHE signal, which produces the symmetric signal, is canceled out by the dynamical spin injections from both interfaces. The origin of the asymmetric is mainly due to the combination between the eddy current and the anisotropic magnetoresistance. Figure 1c shows the resonant magnetic field as a function of the microwave frequency. This dependence was confirmed to be well explained by Kittel’s equation given by

1 $$\begin{aligned} f_0 = \frac{\gamma }{2\pi } \sqrt{\mu _0 H_\mathrm{res}(\mu _0 H_\mathrm{res} + M_S)} \end{aligned}$$

with the gyromagnetic ratio $\gamma /2\pi = 28$ GHz/T and the saturation magnetization $M_S =1.80$ T. This means that the observed signal is caused by the FMR of the CoFeB film^29^.

**Figure 2:**
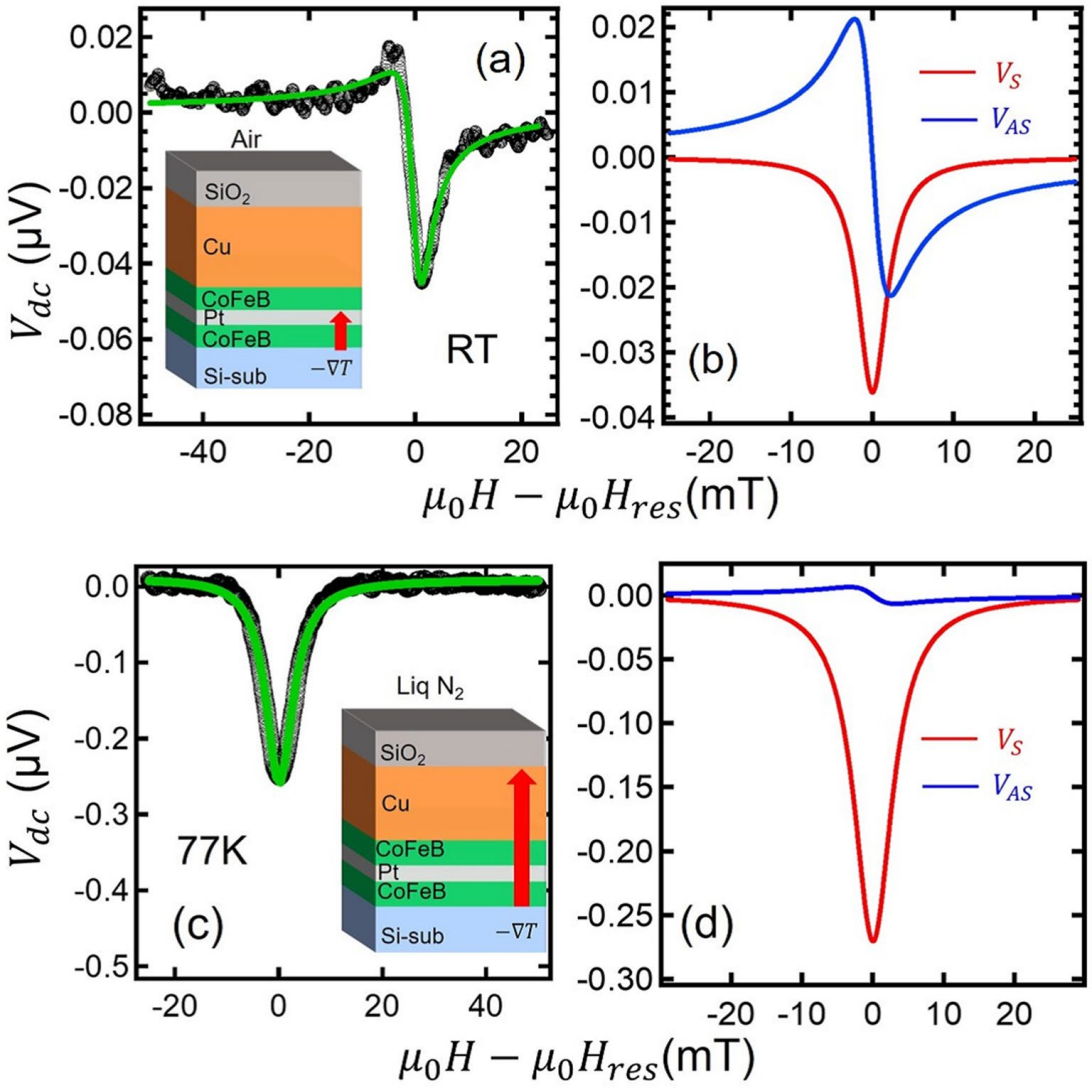
Field dependence of the dc voltage under the microwave with $f=6$ GHz measured at (**a**) room temperature and (**c**) 77 K. The solid lines represent the fitting curve using Eq . (2). Separation of the fitting curve into symmetric (red) and anti-symmetric (blue) components for (**b**) room temperature and (**d**) 77 K.

We now discuss the shape of the voltage spectra. Figure 2a shows the voltage spectra with $$f= $$ 6 GHz. Although the shape of the spectra is almost asymmetric, a small symmetric component has be confirmed. In order to analyze the spectra more quantitatively, we use the following equation^30,31^.

2 $$\begin{aligned} V_\mathrm{tot} (H_\mathrm{ext}) = V_\mathrm{sym} \frac{\Delta H^2}{(H_\mathrm{ext}-H_\mathrm{res})^2+\Delta H^2} + V_\mathrm{asym} \frac{\Delta H (H_\mathrm{ext}-H_\mathrm{res})}{(H_\mathrm{ext}-H_\mathrm{res})^2+\Delta H^2} \end{aligned}$$

Here, $V_\mathrm{sym}$ and $V_\mathrm{asym}$ are, respectively, the amplitude of the symmetric and anti-symmetric components and $\Delta H$ is the linewidth of the FMR peak. As seen in Fig. 2b, the fitting curve well reproduces the observed spectra and $V_\mathrm{sym}$ and $V_\mathrm{asym}$ are estimated as − 0.037 $\mu$V and 0.021 $\mu$V, respectively. Thus, non-negligible symmetric component with the negative sign is obtained. This is because we did not apply the temperature control of the sample intentionally. According to the previous study^19,27,28^, the negative ISHE signal in the present coordinate corresponds to the dynamical spin injection from the upper interface. This indicates that the dynamical spin injection from the upper interface is larger than that from the lower interface. The small amplitude of the symmetric component is due to the cancellation of the dynamical spin injection from both side. We also observed the anti-symmetric field dependence within the same order of the magnitude. As mentioned in the introduction^28^, the origin of the anti-symmetric component is mainly caused by the galvanomagnetic effect due to the direct current flow in the FM, but its quantitative understanding is still controversial issue. Moreover, the similar undesired effects is known to contribute to the symmetric component. However, its magnitude is comparable or smaller than the anti-symmetric component. We emphasize that the clear negative change is observed in the symmetric signal even by subtracting the undesired component. This is a reasonable scenario because the generated heat mainly diffuses from the top to the bottom layers ($-z$ direction) owing to the large thermal sink of the Si substrate.

We have also performed a similar measurement at 77K by immersing the sample in liquid Nitrogen. In this situation, the temperature of the specimen surface is fixed at the liquid Nitrogen temperature. leading to the acceleration of the upward heat-flow. Therefore, we expect the enhancement of the symmetric voltage with the same sign as that at RT. As can be seen in Fig. 2c, we have observed a clear negative symmetric component with the magnitude larger than that at room temperature (RT). This is a surprising fact because the longitudinal resistance of the sample is much smaller than that at room temperature owing to the reduction of the Cu-electrode resistance. Since the induced voltage is proportional to the longitudinal resistance of the sample, this result indicates the significant enhancement of the thermal spin injection efficiency due to the effective increase of the temperature gradient. We also emphasize that the field dependence is well reproduced by Eq. (2) and that the ratio defined by $V_S/V_A$ at 77 K exceeds 50, much larger than that at room temperature, as shown in Fig. 2d. This is also consistent with the enhanced efficiency for the dynamical spin injection at 77 K.

**Figure 3:**
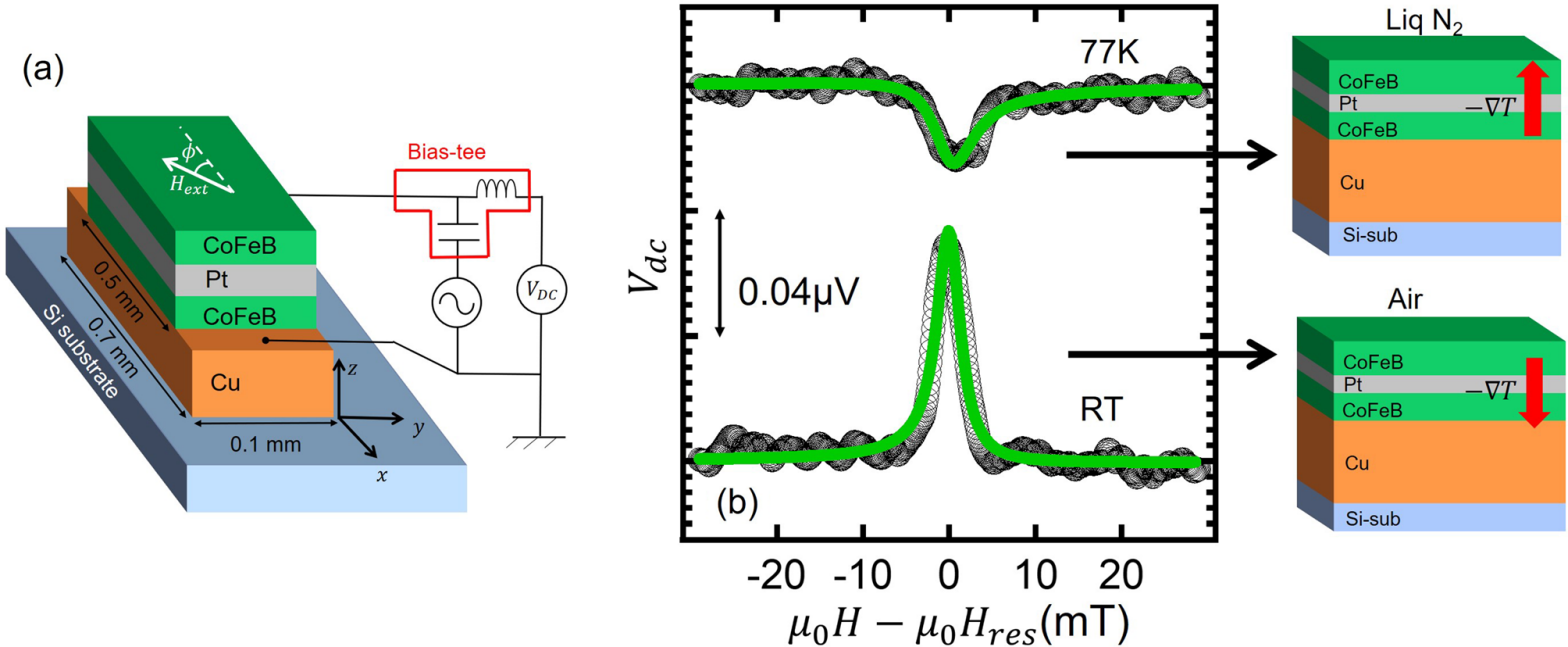
(**a**) Schematic illustration of the CoFeB/Pt/CoFeB trilayer device fabricated on a Cu stripline and the probe configuration for the RF measurement. (**b**) Field dependence of the dc voltage under the microwave with $f=6$ GHz measured at room temperature (bottom) and 77 K (top). The solid lines represent the fitting curve using Eq. (2). The inset show the schematic illustration of the heat flow direction due to the FMR heating for each temperature.

To obtain more definite evidence of the significant contribution of the thermal spin injection, we have prepared a similar trilayer on a Cu strip line, as schematically shown in Fig. 3a. By considering the aforementioned results, the location of the Cu strip line significantly affects the temperature profile in the trilayer^19,24^. In the present structure, at room temperature, the Cu heat sink in between the trilayer and the Si substrate increases the downward heat flow, leading to the enhancement of the negative ISHE signal. On the other hand, at 77K, the upward heat flow decreases, leading to the reduction of the ISHE signal. Figure 3b shows the observed spectra at RT and 77K together with their fitting curves using Eq. (2). The results show good agreement with our expectation. These strongly support that the thermal contribution in the dynamical spin injection is important.

It also should be noted that there are two possible contributions for the thermal spin injection. One is the spin-dependent Seebeck effect driven by the spin-polarized current and the other one is the spin Seebeck effect driven by the magnon spin current. In the metallic structure, the spin-dependent Seebeck effect was naively considered as the major mechanism. However, recent studies indicates the importance of the magnon Seebeck effect in the metallic system^32,33^. Since it is still an important milestone to identify the contribution of each component separately, the dynamical spin injection under the temperature control may solve the key for the quantitative analysis of each contribution Thus, the combination between the spin pumping and the thermal spin injection is a key for the efficient dynamical spin injection.

## Conclusion

We have examined the dynamical spin injection in the CoFeB/Pt/CoFeB trilayer by the electrical detection method using a thick Cu electrode. The efficiency of the dynamical spin injection was significantly reduced because of the symmetric spin pumping from the upper and lower interfaces. However, the finite symmetric component due to the ISHE was found to be induced in the voltage spectra. By changing the temperature gradient in the trilayer, we confirmed that the origin of the ISHE voltage is the thermal spin injection. Our results indicate that the heat control is significant in order to perform the efficient dynamical spin injection.
